# Pilot Study Assessing the Hemodynamic Impact and Post-Exercise Hypotension Induced by High- Versus Low-Intensity Isometric Handgrip in Patients with Ischemic Heart Disease

**DOI:** 10.3390/jcdd12100405

**Published:** 2025-10-12

**Authors:** Giuseppe Caminiti, Matteo Vitarelli, Maurizio Volterrani, Giuseppe Marazzi, Vincenzo Manzi, Valentino D’Antoni, Simona Fecondo, Sara Vadalà, Barbara Sposato, Domenico Mario Giamundo, Alberto Grossi, Valentina Morsella, Ferdinando Iellamo, Marco Alfonso Perrone

**Affiliations:** 1 Department of Human Science and Promotion of Quality of Life, San Raffaele Open University, 00163 Rome, Italy; matteo.vitarelli@uniroma5.it (M.V.); maurizio.volterrani@uniroma5.it (M.V.);; 2 Cardiology Rehabilitation Unit, IRCCS San Raffaele, 00163 Rome, Italy; giuseppe.marazzi@sanraffaele.it (G.M.); valentino.dantoni@yahoo.it (V.D.); fecondosimona@outlook.it (S.F.); vadasara21@gmail.com (S.V.); barbara.sposato@sanraffaele.it (B.S.); valentina.morsella@sanraffaele.it (V.M.); 3 Department of Neurosciences, Biomedicine and Movement, University of Verona, 37134 Verona, Italy; 4 Department of Humanities, Università Telematica Pegaso, 80132 Naples, Italy; vicenzo.manzi@unipegaso.it; 5 Department of Systems Medicine, Tor Vergata University, 00133 Rome, Italy; jamundus20@libero.it; 6 Division of Cardiology, Policlinico Casilino, 00133 Rome, Italy; 7 Division of Cardiology and Sports Medicine, Department of Clinical Sciences and Translational Medicine, University of Rome Tor Vergata, 00133 Rome, Italy; iellamo@uniroma2.it (F.I.); marco.perrone@uniroma2.it (M.A.P.)

**Keywords:** isometric exercise, hypertension, handgrip, ischemic heart disease

## Abstract

**Background**: Isometric handgrip (IHG) exercise reduces blood pressure (BP) in both normotensive and hypertensive individuals. However, there are few studies specifically addressing its effects in hypertensive patients with ischemic heart disease (IHD). This research aimed to compare acute hemodynamic responses and post-exercise hypotension to single bouts of IHG handgrip performed at two different intensities in patients with IHD. **Methods**: Fifty-four sedentary patients were enrolled and randomly assigned to one of three groups: (1) high-intensity isometric handgrip performed at 70% of maximal voluntary contraction (MVC) (IHG-70%); (2) low-intensity isometric handgrip performed at 30% of MVC (IHG-30%); (3) control group (no exercise). Heart rate and BP were measured, and transthoracic echocardiography was performed at baseline, during exercise (lasting 3 min), and after 15 min post-exercise. BP was also measured at 30, 45, and 60 min of recovery. **Results**: No significant changes in systolic BP occurred during the exercise phase between the three study groups. Systolic BP decreased significantly in IHG-70% compared to the control at 30 (−7.7 ± 1.9; *p* = 0.035) and 45 min (−8.1 ± 2.3; *p* = 0.021) post-exercise, while there were no significant differences between IHG-70% and IHG-30% at different time-points. There were no significant changes in diastolic BP between the two active groups and between IHG-70 and IHG-30 versus control at different time-points (repeated-measures ANOVA *p* = 0.257). Global work efficiency was unchanged in IHG-70% (−4%) and IHG-30% (+1%) compared to control (ANOVA *p* = 0.154). **Conclusions**: High-intensity and low-intensity isometric handgrip exercises did not cause hemodynamic impairment in IHD. High-intensity exercise was more effective than low-intensity in reducing post-exercise systolic BP.

## 1. Introduction

The vast majority of patients with ischemic heart disease (IHD) have a history of hypertension and take several drugs for maintaining blood pressure (BP) values within the normal range [1]. Achieving optimal BP control in patients with IHD has a positive impact on their prognosis [2], and it is among the most important pursued goals during cardiac rehabilitation programs [3]. Physical activity alongside other non-pharmacological treatments is a well-established intervention for preventing the rise of BP in pre-hypertension and for lowering BP in subjects with hypertension [4,5]. While all types of exercise have been shown to reduce BP, continuous aerobic exercise has the most robust evidence supporting its BP-lowering effects [6,7]. Isometric exercise (IE) has been shown to effectively reduce both systolic and diastolic BP with effects comparable to those of aerobic training [8,9]. This modality may be particularly advantageous in the management of hypertension among older adults, as it can be safely performed even by patients with limited functional capacity or those who have contraindications to aerobic activity [10]. Additionally, IE offers a time-efficient option, as short sessions lasting 15–20 min have demonstrated significant benefits in BP reduction [11]. The use of IE for the treatment of hypertension in patients with IHD has been scarcely investigated to date. In fact, concerns about causing an excessive increase in systolic BP and hemodynamic overload of the left ventricle (LV) have prevented the use of IE in this population. It has been demonstrated that the increase in blood pressure (BP) during isometric exercise is directly proportional to both the amount of muscle mass engaged and the intensity of the contraction. Consequently, different isometric exercises elicit distinct effects on BP and hemodynamic parameters. In a recent study involving patients with IHD, low-intensity bilateral knee extension was associated with a significant rise in systolic BP and a marked reduction in myocardial work efficiency. In contrast, low-intensity isometric handgrip (IHG) produced only minor changes in BP and exhibited neutral hemodynamic effects, with no significant impact on left ventricular contractile efficiency [12]. While low-intensity IHG appears to be a suitable IE modality for IHD patients in terms of tolerability and safety, it still remains to be established if it is also effective in reducing BP in this population. An alternative approach could involve performing IHG at high intensity, which appears to be justified by evidence showing an intensity-dependent reduction in resting blood pressure in hypertensive and pre-hypertensive subjects [13,14]. A recent study demonstrated that IHG performed at 60% of maximal voluntary contraction (MVC) was more effective in reducing both systolic and diastolic BP compared to IHG performed at 30% MVC in healthy males [15]. The effectiveness and safety of high-intensity IHG in a population of hypertensive patients with IHD has not been tested yet. The assessment of LV myocardial work through speckle tracking echocardiography is an accurate, non-invasive technique for evaluating LV contraction efficiency [16], and it has been used for investigating the effects of exercise on myocardial function [17]. In the present study, we compared the hemodynamic responses and post-exercise BP effects elicited by single bouts of IHG exercise performed at high versus low intensity. We hypothesized that IHG performed at 70% of MVC would be as safe as IHG performed at 30% MVC, while being more effective in reducing post-exercise BP. The primary endpoint was changes in systolic BP during the post-exercise recovery phase. Secondary endpoints included changes in LV myocardial work efficiency during the exercise and diastolic BP during the post-exercise phase.

## 2. Materials and Methods

### 2.1. Population

A total of 56 male and female patients involved in secondary prevention and cardiac rehabilitation programs at San Raffaele IRCCS of Rome were enrolled in the study. Inclusion criteria were age over 50 years; diagnosed hypertension; history of IHD encompassing prior acute coronary syndrome (ST-elevation myocardial infarction (STEMI), non-ST-elevation myocardial infarction (NSTEMI, or unstable angina), percutaneous coronary intervention (PCI), or coronary artery bypass grafting (CABG); stable clinical conditions (no hospitalizations in the past 6 months and unchanged pharmacological therapy for at least 3 months); physically active, whereby patients were considered exercisers if a planned and structured physical activity was performed for at least 30 min on three days or more during the past three months [18]. Exclusion criteria included signs or symptoms of myocardial ischemia or complex ventricular arrhythmias at rest or during exercise testing; incomplete revascularization; permanent or recurrent atrial fibrillation; resting BP ≥ 160/100 mmHg despite treatment; significant valvular disease; hypertrophic cardiomyopathy; having an history of chronic heart failure or having signs and/or symptoms of heart failure; having an echocardiography LV ejection fraction below 40%. Additional exclusions included extracardiac conditions such as anemia (blood hemoglobin levels < 10.5 g/dL), advanced COPD (with GOLD stage III–IV), and symptomatic peripheral artery disease (Leriche–Fontaine stage II–IV) and a poor acoustic window: patients were considered ineligible if the echocardiographic images were not of sufficient quality to allow clear identification of the left ventricular and left atrial borders, thereby precluding speckle-tracking analysis. The study was approved by the Ethics Committee of San Raffaele IRCCS (Protocol No. 28/2024), and all participants provided written informed consent in accordance with the Declaration of Helsinki. The recruitment period started in October 2024 and was concluded in March 2025.

### 2.2. Study Design

**Figure 1:**
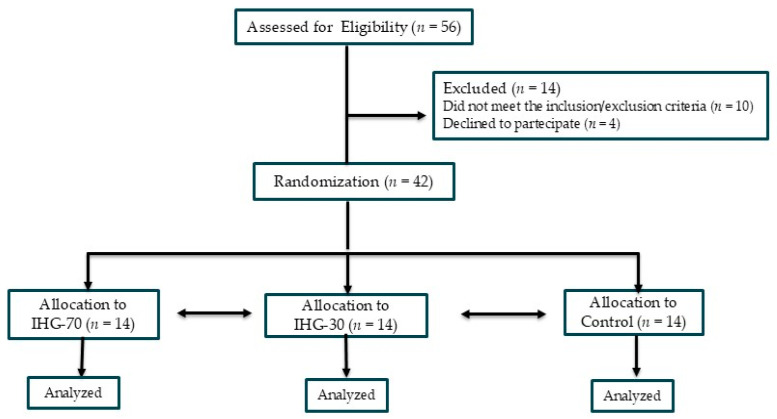
Flow chart of the study.

Figure 1 summarizes the study flow chart. This research was conceived as a three-armed parallel randomized pilot study in which patients were randomly assigned in a 1:1:1 ratio to one of three study groups: (1) high-intensity IHG in which IE was performed at 70% of MVC (IHG-70); (2) low-intensity IHG in which IE was performed at 30% of MVC (IHG-30); (3) control with no exercise. Randomization was performed using a computer-generated sequence of random numbers, created prior to patient enrollment. To ensure that study personnel and participants were unaware of group assignment at the time of enrollment, allocation was concealed by means of sequentially numbered sealed opaque envelopes that were opened only after a patient’s eligibility was confirmed. This was a single-blinded study in which the sonographers who performed the offline analysis of echocardiographic parameters were blinded to patient group allocation. The initial assessment (Visit 1) included the following: collection of clinical history; measurement of resting BP and heart rate; anthropometric measurements (weight and height); a symptom-limited ergometric test. The ergometric test was conducted on a cycle ergometer (Quark CPET, COSMED, Frascati, Italy) using a ramp protocol with 20-watt increments every 2 min. Patients who met the inclusion and exclusion criteria were invited to participate in the study and provided written informed consent. A second evaluation (Visit 2) was conducted within one week of Visit 1. During Visit 2, participants were familiarized with the device used in the experimental session. The experimental session for each participant was completed within one week following Visit 2.

### 2.3. Experimental Sessions

All experimental sessions were performed in the morning, between 8:30 am and 12 am. Patients were required to avoid consuming coffee and smoking cigarettes for at least 12 h before the sessions. A light breakfast was allowed at least 2 h before the session. Patients were allowed to take their regular morning medications on the day of the session. The experiments were carried out with participants lying on a bench, positioned with their backs reclined at a 120-degree angle from the horizontal. A sonographer was positioned on the participant’s left side, and a sphygmomanometer cuff (GIMA, S.p.A., Gessate [MI], Italy) was placed on the arm opposite the dominant one used for handgrip exercises. The MVC was determined by having each participant perform three maximal handgrip efforts, each lasting 3–5 s, with 1-min rest intervals between attempts. Exercise intensity was then set at 30% of MVC for the low-intensity group and at 70% for the high-intensity group. The isometric exercise phase lasted 3 min, during which participants were instructed to maintain a constant force. To avoid Valsalva maneuvers, they were guided to breathe normally, maintaining a steady rhythm and depth. Echocardiographic imaging began after the first minute of exercise. The sonographer remained on the participant’s left side, and BP was measured via a cuff on the right arm. Echocardiographic acquisitions and BP measurements were performed at three time-points: at rest; during the isometric exercise, beginning 1 min after exercise onset; and 15 min after the exercise ended. BP measurement but no echocardiography was also carried out at 30 and 45 and 60 min after completion of the isometric effort. For the control group, the same measurement timeline was followed, but participants remained at rest for the entire duration of the session. They were also positioned supine on a bench with a 120-degree recline, mirroring the setup used for the intervention groups.

### 2.4. Echocardiography

All echocardiographic assessments were conducted using the Vivid E95^®^ cardiovascular ultrasound system (GE Healthcare, Chicago, IL, USA) equipped with a 4.0 MHz transducer, throughout the duration of the study. Examinations were performed with single-lead ECG monitoring, and image acquisition followed the recommendations of the European Association of Cardiovascular Imaging [19]. All images were digitally archived for offline analysis. Strain analyses were carried out offline by two trained technicians that used a proprietary software (EchoPAC, version 10.8; GE Vingmed Ultrasound, Horten, Norway) and who were blinded to patient group allocation. Left ventricular (LV) diastolic function was evaluated using the E/A ratio, calculated as the ratio between early (E-wave) and late (A-wave) diastolic filling velocities. Color tissue Doppler imaging was performed in the apical four-chamber view, with the sample volume positioned at the lateral mitral annulus. The E/e’ ratio was defined as the ratio between E-wave velocity and the average of septal and lateral e’-wave velocities. Left atrial (LA) volume was measured from the apical four-chamber and two-chamber views at end-systole, before mitral valve opening, using the biplane Simpson’s method. LA volume index (LAVI) was calculated by dividing the LA volume by the body surface area. LV end-diastolic volume (LVEDV) and end-systolic volume (LVESV) were measured from apical four- and two-chamber views, and the LV ejection fraction (LVEF) was computed using the modified Simpson’s method. Stroke volume (SV) was calculated as LVEDV minus LVESV, and cardiac output (CO) was derived as the product of SV and heart rate. Ejection fraction was also expressed as EF = (EDV − ESV)/EDV. The global longitudinal strain (GLS) of the LV was measured from apical four-chamber, three-chamber, and two-chamber views. The software automatically tracked endocardial borders and computed the peak negative systolic strain value in each segment, which represented maximum contractility. Manual corrections were made as needed to optimize contouring. The overall LVGLS was determined as the average of all segmental values. LA strain analysis was conducted using apical four-chamber and two-chamber views. The software automatically traced both endocardial and epicardial borders using R-R gating, with the R-wave as the reference point. If necessary, manual adjustments were applied. Control points were placed along the central myocardial curve to define the region of interest. Longitudinal strain curves were generated for each segment, and average segmental curves were computed. Reservoir, conduit, and contractile LA strain components were derived from the full strain curve. Myocardial work was assessed during the period between mitral valve closure and opening. Peak atrial contraction strain (PACS) was defined as the positive peak strain occurring at the onset of LV diastole, before atrial contraction; peak atrial longitudinal strain (PALS) was defined as the positive peak occurring during LV systole, at the end of atrial diastole. A 17-segment polar (bull’s-eye) plot was generated to represent regional and global myocardial work. The global work index (GWI) was calculated as the total area under the work curve between mitral valve closure and opening. Global constructive work (GCW) included positive work during systolic shortening and negative work during isovolumetric relaxation lengthening. Global wasted work (GWW) was defined as work performed during inappropriate shortening in isovolumetric relaxation and lengthening during systole. Global work efficiency (GWE) was calculated as the ratio of GCW to the sum of GCW and GWW. Timing of valvular events was determined using pulsed-wave Doppler at the levels of the mitral and aortic valves and confirmed through 2D imaging in long-axis and apical views [20].

### 2.5. Statistical Analysis

This study was conceived as a pilot investigation; accordingly, no formal a priori sample size calculation was performed specifically to detect differences between the two active intervention groups. Nonetheless, we hypothesized that at least one of the interventions would reduce systolic blood pressure compared with control. To guide sample size estimation, we referred to effect sizes reported in previous trials evaluating the impact of isometric handgrip exercise versus control on blood pressure [21,22]. Based on these data, we estimated that detecting a 6.5 mmHg reduction in systolic blood pressure, with an assumed standard deviation of 6.0 mmHg, across three groups (two intervention arms and one control), with α = 0.05 (Bonferroni-adjusted to 0.025 for two planned comparisons) and 80% power, would require 14 participants per group, corresponding to a total sample size of 42 participants. Continuous variables are presented as the mean ± standard deviation (SD). The assumption of normality was assessed using the Shapiro–Wilk test. Unadjusted group differences were evaluated using one-way ANOVA, and adjusted comparisons were conducted using ANCOVA with prespecified covariates. To evaluate changes in variables across the different phases of the experimental sessions, a repeated-measures two-way ANOVA was applied with Bonferroni correction used for post hoc comparisons. A *p*-value of less than 0.05 was considered statistically significant. Categorical variables were expressed as absolute frequencies and percentages and compared using the chi-square test. All statistical analyses, data processing, and graphical presentation were performed using IBM SPSS Statistics, version 26.0.

## 3. Results

The study enrolled 56 patients; of these, 8 were excluded due to excessively high BP values, 2 for having a poor acoustic window and 4 withdrew consent, refusing to continue participation in the study. In total, 42 patients were therefore randomly assigned to one of the three study groups, with each group comprising 14 patients. All patients randomized completed the study and their data were analyzed. Baseline clinical features of the patients are reported in Table 1.

**Figure 2:**
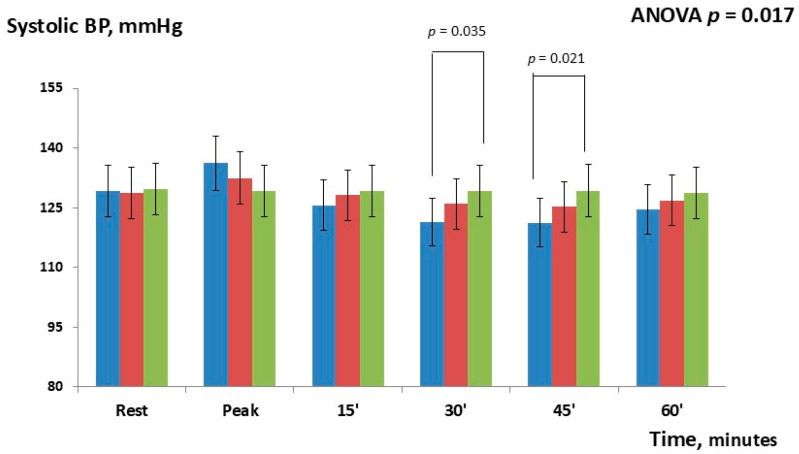
Changes in systolic BP observed during and after the three experimental sessions: IHG-70 (blue bars), IHG-30 (red bars), control (green bars). During the post-exercise phase, systolic BP significantly decreased between IHG-70 and control at 30 min and 45 min. The statistical analysis was performed by repeated-measures ANOVA and Bonferroni’s post hoc tests.

At baseline, the three groups were comparable regarding age, anthropometric parameters, rate of comorbidities, and pharmacological therapy. Sensitivity analyses using ANCOVA, performed to account for potential clinical differences in baseline parameters across the three groups, confirmed the findings of the initial ANOVA and did not reveal any significant between-group differences. The average number of medications taken for lowering BP was 2.8 ± 0.8. All patients analyzed had a previous myocardial infarction that was STEMI (80.9%): anterior STEMI—61.6%; inferior STEMI—38.4%. In total, 26 out of 42 (61.9%) were overweight and 13 out of 42 (30.9%) were obese. Experimental sessions were well tolerated and no symptoms occurred during the exercise and the recovery phases. Changes in systolic BP occurring at different time-points are shown in Figure 2 and Table 2.

**Figure 3:**
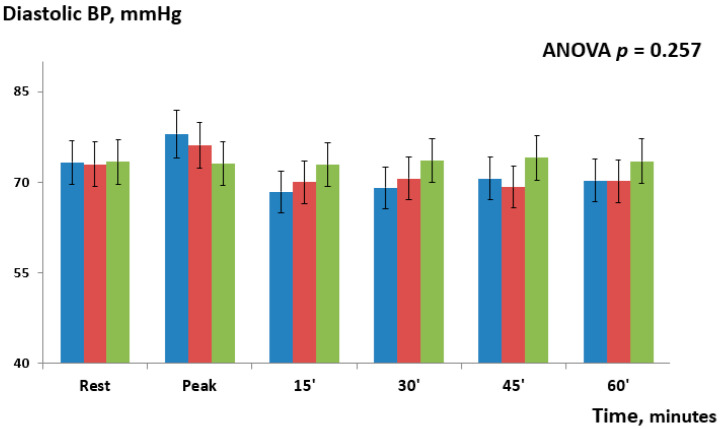
Changes in diastolic BP observed during and after the three experimental sessions: IHG-70 (blue bars), IHG-30 (red bars), control (green bars). There were no significant differences in diastolic BP between the two active groups and between IHG-70 and IHG-30 versus control at different time-points. The statistical analysis was performed by repeated-measures ANOVA.

Overall, the repeated-measures ANOVA showed significant changes between the three study groups (*p* = 0.017). During the exercise phase, systolic BP modestly increased in both IHG-70 [+7.0 mmHg (95% CI = 4.8–9.6), *p* = 0.065] and IHG 30 [+3.2 mmHg (95% CI = 1.4–7.3); *p* = 0.231] compared to control; post hoc Bonferroni tests did not show significant differences between the two active groups or versus the control. During the post-exercise phase, post hoc Bonferroni tests showed that systolic BP significantly decreased between IHG-70 and control at 30 min [−7.7 mmHg (95%CI= −5.6–−9.1); *p* = 0.035] and 45 min [−8.1mmHg (95% CI = −6.3–−9.9); *p* = 0.021]. There were no significant changes between the two active groups with the width differences observed at 30 min [−4.5 mmHg (95% CI = −2.6–−6.5); *p* = 0.084] and between IHG-30 and the control. Changes in diastolic BP occurring at different time-points are shown in Figure 3 and Table 2.

There were no significant differences in diastolic BP between the two active groups or between IHG-70 and IHG-30 versus the control (repeated-measures ANOVA *p* = 0.257). There were no significant changes in GCW (ANOVA *p* = 0.372) and GWW (ANOVA *p* = 0.298) between the two active groups or between the active groups and the control. There were no significant changes in GWE between the two active groups or between the active groups and the control at different time-points (ANOVA *p* = 0.154,). The E/e’ ratio, PALS, and PACS were unchanged in all groups.

No significant changes occurred between IHG-70 and IHG 30 or between IHG-30 and control at different time-points.

## 4. Discussion

The addition of daily short sessions of isometric exercise (IE) as an adjunctive strategy for the long-term management of hypertension in patients with ischemic heart disease (IHD) who are already receiving anti-hypertensive therapy appears to be a promising approach. However, it remains unclear which type of IE is most suitable for this population, specifically, one that combines minimal hemodynamic impact during execution with a clinically significant post-exercise BP reduction. The present study comparatively evaluated the acute hemodynamic responses and post-exercise BP-lowering effects of single bouts of IHG performed at high versus low intensity. We found that both intensities elicited only modest increases in BP and did not produce significant detrimental hemodynamic changes during the exercise phase. However, only the higher-intensity protocol induced a significant post-exercise BP reduction compared with control. Proposed mechanisms for explaining the BP reduction during the post-exercise phase include the following: stimulation of mechano- and metaboreceptors by metabolite accumulation during isometric exercise, reduced sympathetic outflow, reactive hyperemia [23,24,25]. Although the design of the present study does not allow for direct investigation of the pathophysiological mechanisms underlying the blood pressure reduction induced by IHG, it can be hypothesized that the aforementioned mechanisms may be amplified when the exercise is performed at higher intensity. Since the literature on the BP effects of high-intensity IHG is very limited, and the available studies have been conducted exclusively in healthy individuals, the present study provides novel evidence with potential clinical implications. Specifically, the favorable hemodynamic tolerability observed during IHG-70, together with the post-exercise BP reduction, supports the consideration of this modality as a potential exercise option for BP management in hypertensive patients with IHD.

### 4.1. Hemodynamic Tolerability

In this study, systolic BP increases during both IHG-30 and IHG-70 were modest and left ventricular (LV) myocardial work efficiency remained unchanged. These findings are consistent with previous observations in hypertensive older women [26] and in patients with IHD [12]. However, this is the first study assessing the impact of high-intensity IHG on LV myocardial work and efficiency. The finding that even high-intensity IHG did not induce significant hemodynamic effects suggests that this mode of IE is as safe as low-intensity IHG for patients with hypertension and IHD. This research showed no changes in metrics of diastolic function such as the E/e’ ratio and PALS during the isometric efforts. This result is probably due to the modest increase in afterload observed during both IHG-30 and IHG-70 and it can, as a first hypothesis, be attributed to the effectiveness of the pharmacological therapy received by the enrolled patients; however, it could also be related to the training status of the patients. In the study of Rovithis et al. [27], who compared changes in diastolic function during IHG in physically active versus sedentary men, the group of active men did not show a statistically significant change in the E/e’ ratio in response to IHE, while the inactive participants’ E/e’ ratio was higher at peak activity in the IHG. The findings of this research differ from those of Javidi et al. [15], who reported a significant systolic BP rise during high-intensity IHG. This discrepancy may be attributable to differences in study populations; in our cohort, patients were already receiving multiple antihypertensive agents which may have attenuated the BP response during the isometric effort.

### 4.2. Post-Exercise Hypotension

In this study, a significant reduction in systolic BP was observed only after IHG performed at high intensity. This is a unique finding in the population of IHD patients that needs to be confirmed and expanded. Regarding BP reduction in the post-exercise phase, the results of the present study comply with the study of Javidi et al. [15], who observed that high-intensity IHG evoked a greater decrease in systolic BP compared to low-intensity IHG (−15.5 mmHg versus −5.0 mmHg, respectively). On the contrary, BP did not decrease after IHG-30. The effects of low-intensity IHG on BP are controversial: despite it having been demonstrated that a single bout of low-intensity IHG reduces BP during the recovery phase in pre-hypertensive and hypertensive patients [28,29,30], such findings have not been consistently confirmed [26,31]. This variable response can be expected since the BP response to IE is influenced by several factors including age, gender, comorbidities, and the number of medications taken [32]. In the review of Loaiza-Betancur et al. [33], low-intensity IHG decreased systolic BP of 5.4 mmHg overall; however, it was more effective in subjects aged under 45 years. Similarly, in a recent study of young physically active men, Melnikov et al. [34] documented significant BP reduction after IHG performed at 20% of MVC. Instead, the results obtained in the present study suggest that low-intensity IHG is not capable of significantly reducing post-exercise BP, at least in elderly patients with IHD also carrying other comorbidities and who were receiving, on average, more than two antihypertensive medications. We think that the present study adds useful information regarding the choice of exercise modality and intensity aimed at the non-pharmacological management of hypertension in patients with IHD. Moreover, it emphasizes the utility of performing a non-invasive echocardiography assessment of the hemodynamic response before starting an IE protocol. Intensities were characterized by a mild comparable hemodynamic response.

### 4.3. Limitations

The main limitation of the present study is the small sample size that did not allow us to reveal significant differences in the BP-lowering effect between the two active groups. Therefore, adequately powered future investigations will be required to determine whether high-intensity IHG is more effective than low-intensity IHG in reducing BP. However, it should be noted that since this study was powered enough to reveal differences between the active groups and the control, its result can be considered reliable. The measurement of BP during the post-exercise phase was very short; therefore, the duration of the hypotensive effect induced by high-intensity IHG exercise remains undetermined. Future research should ideally incorporate 24 h ambulatory blood pressure monitoring in order to more precisely characterize the duration of the antihypertensive effect of isometric exercise. Since in each group more than 75% of the enrolled patients were male, the female sex was underrepresented; therefore, caution is warranted when extrapolating the results to women. The results of this study were obtained from a single bout of IHG, during which patients were instructed to maintain a constant contraction for three minutes. It cannot be excluded that alternative protocols might elicit different BP responses. Caution in interpreting the data is needed since strain echocardiography has several technical limitations [35,36]; therefore, further confirmations of our results should also be obtained with alternative diagnostic techniques such as, for example, cardiac magnetic resonance imaging. In this study, we did not test the reproducibility of echocardiography measurements; however, we utilized data from our previous research in which we assessed the reproducibility of all echocardiography measurements and in which we used the same echocardiographic device as in the present study [37]. A further limitation of the present study is the absence of continuous ECG monitoring during the experimental sessions. Although a three-lead ECG was recorded during echocardiographic assessments for technical purposes (i.e., image synchronization), no continuous cardiac rhythm monitoring was performed throughout the isometric handgrip protocol. As such, transient arrhythmias or subtle autonomic fluctuations may have gone undetected. However, no adverse symptoms or clinical signs were observed during or after the exercise sessions.

## 5. Conclusions

In the present study, both low- and high-intensity IHG exercises were hemodynamically well tolerated by patients with IHD but only high-intensity IHG induced a significant post-exercise systolic BP reduction. The use of high-intensity IHG appears to be a safe and effective non-pharmacological intervention for reducing BP in IHD patients. Further studies should assess whether it is more effective than low-intensity IHG in reducing BP.

## Figures and Tables

**Table 1:** Baseline features of recruited patients according to the three groups’ allocation.

	IHG-70 (n = 14)	IHG-30 (n = 14)	Control (n = 14)	ANOVA *p*
Age, y	66.4 ± 11.2	65.9 ± 12.7	66.1 ± 12.0	0.571
BMI, kg/m^2^	28.4 ± 7.3	28.3 ± 9.1	27.9 ± 6.6	0.796
Males, n (%)	11 (78.6)	11 (78.6)	11 (78.6)	0.457
Previous STEMI/NSTEMI-UA, n (%)	12 (85.0)/2 (14.0)	12 (85)/2 (14.0)	10 (71.4)/4 (28.6)	0.642
Previous CABG/PCI, n (%)	7 (50.0)/11 (78.5)	9 (64.2)/10 (71.4)	10 (71.4)/11 (78.6)	0.966
Multivessel disease, n (%)	12 (85.7)	12 (85.7)	10 (71.4)	0.851
Carotid artery disease, n (%)	7 (50.0)	6 (42.8)	6 (42.8)	0.598
Diabetes, n (%)	4 (28.6)	6 (43.0)	5 (20.0)	0.883
Hypercholesterolemia, n (%)	15 (100.0)	14 (93.3)	15 (100.0)	0.248
eGFR, mL/min per 1.73 m^2^	78.3 ± 11.6	67.4 ± 12.1	75.7 ± 13.8	0.089
SBP, mmHg	126.6 ± 34.0	125.8 ± 27.4	126.1 ± 30.9	0.144
DBP, mmHg	82.2 ± 14.7	83.6 ± 14.4	82.8 ± 15.3	0.132
HR, b/min	62.1 ± 9.5	60.8 ± 11.0	63.5 ± 8.9	0.091
NT-proBNP, pg/mL	71.4 ± 10.8	68.3 ± 12.4	66.2 ± 9.2	
Echocardiography				0.527
LVEDV, mm^3^/m^2^	64.2 ± 22.3	65.0.9 ± 15.4	68.7 ± 18.8	0.232
LVESD, mm^3^/m^2^	36.7 ± 12.3	37.1 ± 14.6	37.6 ± 13.7	0.522
LVEF, %	55.1 ± 8.1	53.9 ± 8.2	54.5 ± 10.7	0.385
LV GLS, %	−16.9 ± 4.3	−17.3 ± 4.8	−17.0 ± 5.3	0.077
E/E’	8.6 ± 2.3	8.2 ± 2.9	8.4 ± 2.5	0.491
LAVI, mm^3^/m^2^	29.3 ± 7.1	28.6 ± 6.9	28.8 ± 7.7	0.287
MR mild/moderate, n (%)	9 (64.3)/5 (35.7)	10 (71.5)/4 (28.5)	11 (78.6)/3 (21.4)	0.182
PALS. %	15.7 ± 3.6	16.2 ± 3.3	16. ± 3.1	0.362
PACS, %	13.2 ± 2.9	12.9 ± 3.0	13.2 ± 3.7	0.371
Treatment				
ACE-i/ARBs, n (%)	14 (100.0)	15 (100.0)	13 (93.5)	0.441
Betablockers, n (%)	14 (100.0)	13 (93.5)	14 (100.0)	0.642
MRAs, n (%)	4 (28.5)	4 (28.5)	3 (21.4)	0.238
Thiazide diuretic, n (%)	6 (42.8)	7 (50.0)	7 (50.0)	0.692
Ivabradine, n (%)	2 (14.3)	-	1 (7.1)	0.383
Statins, n (%)	14 (100.0)	14 (100.0)	13 (92.8)	0.754
CCAs, n (%)	8 (57.1)	9 (64.3)	7 (50.0)	0.648
Acetylsalicylic acid	13 (92.8)	14 (100.0)	14 (100.0)	0.132
Clopidogrel	3 (21.4)	2 (14.3)	-	0.167

**Table 2:** Comparison of changes in systolic and diastolic BP observed at different time-points. Statistical analysis made by repeated-measures ANOVA.

	Δ Systolic BP		
Timing	IHG-70 vs. IHG-30	IHG-70 vs. Control	IHG-30 vs. Control
Rest	0.6 ± (0.4)	−0.4 ± (0.6)	1.0 ± (1.1)
Peak	3.8 ± (1.8)	7.0 ± (2.1)	3.2 ± (1.6)
15	−2.5± (0.9)	−3.6 ± (1.4)	−1.1 ± (0.6)
30	−4.5 ± (2.9)	−7.7 ± (1.9) *	−3.2 ± (1.9)
45	−4.0 ± (2.6)	−8.1 ± (2.3) *	−4.1 ± (2.2)
60	−2.2 ± (1.3)	−4.1 ± (1.7)	−1.9 ± (1.3)
	**Δ Diastolic BP**		
Timing	IHG-70 vs. IHG-30	IHG-70 vs. control	IHG-30 vs. control
Rest	−0.3 ± (0.6)	−0.1 ± (0.3)	−0.4 ± (0.7)
Peak	1.8 ± (1.1)	4.9 ± (2.8)	3.1 ± (0.9)
15	−1.6 ± (0.4)	−4.5 ± (3.1)	−2.9 ± (1.1)
30	−1.5 ± (1.2)	−4.5 ± (2.7)	−3.0 ± (1.4)
45	1.4 ± (0.7)	−3.5 ± (1.8)	−4.9 ± (2.6)
60	0.1 ± (0.5)	−3.2 ± (1.1)	−3.3 ± (1.5)

* = Bonferroni *p* < 0.05 vs. control.

## Data Availability

The data presented in this study are available upon request from the corresponding authors.

## Abbreviations

The following abbreviations are used in this manuscript:

IHG	Isometric handgrip
BP	Blood pressure
IHD	Ischemic heart disease
MVC	Maximal voluntary contraction
IE	Isometric exercise
LV	Left ventricle
PCI	Percutaneous coronary intervention
CABG	Coronary artery bypass grafting
LA	Left atrial
LAVI	LA volume index
LVEDV	LV end-diastolic volume
LVESV	LV end-systolic volume
LVEF	LV ejection fraction
SV	Stroke volume
CO	Cardiac output
GLS	Global longitudinal strain
PACS	Peak atrial contraction strain
PALS	Peak atrial longitudinal strain
GWI	Global work index
GCW	Global constructive work
GWW	Global wasted work
GWE	Global work efficiency
SD	Standard deviation
